# Electro-Physiology

**Published:** 1878-01

**Authors:** A. Means


[From the Augusta Medical Journal of 1848.]

### ELECTRO-PHYSIOLOGY.

### THOUGHTS UPON THE ADAPTATION OF THE ANIMAL ORGANISM IN THE GENERATION AND MAINTENANCE OF THE ELECTRO-VITAL CURRENTS, ETC.

By A. MEANS, A.M., M.D., D.D.

(*Continued from December number of this Journal.*)

According to the recently expressed views of the Professor of Pisa, the muscular currents, whose existence has been demonstrated, depend for their origin upon "the electric conditions which are produced by the chemical actions of the nutrition of the muscle"—the muscular fibre being regarded as the morphological element whose transformation, effected by the contact of the oxygenized blood, evolves molecular currents, which arising from a multitude of similar points united by a good conductor, and communicating with other points which have not undergone similar metamorphosis, may originate general currents. In this view, the "blood charged with oxygen, and the muscular fibre" become the elements of a pile, i. e. "the liquid and zinc."

But in the complicated organism of the human body, it is neither philosophical, nor necessary to the maintenance of the views which we urge, to limit the generation of electrical currents to one class of causes alone, and we are therefore warranted to admit their formation from any structural arrangement or vital action in the system, competent to produce them. This leads us to notice another law of our fluid.

3d. *It is known to be generated directly under the dominion of the vital principle, and by the exclusive agency of the living tissues, as in the torpedo and gymnotus electricus of Surinam, and to manifest all the striking results found from its action in the inorganic kingdom, as shocks, sparks, evolution of heat, chemical decomposition, deflection of a galvanometer and magnetism.*

The electro-physiological phenomena exhibited by these animals have utterly exploded the opinion that the presence of metals was indispensable to the production of voltaic electricity. Indeed, additional evidence of the fallacy of this dogma has been found in the electrical results which have followed from the lamellar arrangement of charcoal and plumbago; while by combinations of moist masses of animal and vegetable structure, as muscle and brain, beet root and wood, etc., the interesting truth has been established that, under given conditions, the organic kingdoms possess independent electromotive power.

Some valuable facts here call for our attention, derived from the recent critical and accurate experiments of Prof. Faraday, upon the Surinam eel, and from the report upon the voltaic power of the torpedo, made to the Academy of Sciences at Paris a few years since. First, that the membranous and columnar arrangement of their electric organs generate and retain large quantities of electricity, notwithstanding the diffused moisture of the whole interior and adjacent surfaces, and in defiance of the good conducting medium in which they move, which, in the case of inorganic generators, would lead to the speedy dissipation of the current, but here, as in the case of chemical affinities acting under the authoritative supremacy of the vital principle, we are furnished with another instance of complete and essential subordination of ordinary laws to the modifying and controlling powers of life.

Second. In the language of the report, "that the electricity, which produces the discharge, proceeds from the best lobe of the brain, and is transmitted by the nerves to the brain"—for in the experiments of the examining committee, the nerves, when tied, were found promptly to check the flow of the current, indicating, as they say, "in the nerve a peculiar molecular disposition for the transmission of the fluid," an opinion which finds support in the fact that the uninjured nervous trunks and filaments of every animal heretofore submitted to experiment have furnished it an instantaneous passage throughout the line of communication, but when in any instance bisected or crushed, refusing to convey it beyond the breach or laceration.

But again, within the present year, Prof. Liebig, in prosecuting his "investigation of the constituents of the animal fluids which are found without the blood and lymphatic vessels," has succeeded in demonstrating the existence of "free lactic and phosphoric acid in the whole organism, wherever muscle is found;" and what is still more strangely true, but corroborative of the views here presented, "in the animal organism, acids and alkalis are found separated by a membrane, constituting myriads of little galvanic circles, which, as such, must produce chemical and electrical effects." So that the long-repudiated doctrine of the acidity and alkalinity of the animal fluids, taught with so much eclat 190 years ago by the popular professor of the Leyden University, De le Boe Sylvus, however in that age misapprehended and misapplied, seems not to have been entirely chimerical.

Whether, however, this view be accepted or rejected, recent anatomical investigations, as reported by modern micogrophers, have disclosed the interesting fact that the construction of muscular fibre is almost exactly analagous to the electrical organs of fishes, consisting of longitudinal striæ and transverse discs, the latter being "perfectly rectilinear and the included spaces perfectly rectangular," and in the language of Matteucci, "it is very remarkable that with respect to the general circumstances of the two phenomena, the same laws preside over the discharge of electrical fishes and muscular contraction."

4th. *Inorganic currents pass with immense velocity along an unbroken conductor, and with greatest intensity when defended from lateral escape by the investment of a* NON-CONDUCTOR, *as silk or cotton thread, varnish, etc.*

The nervous tissue constitutes, unquestionably, the grand conductor or pathway along which this etherial agent effects its instantaneous and invisible passage to the remotest organs of the system, and the neuri-lemma or unctious theca which invests the tissue in its trunks and thousand ramifications, is, as we regard it, but a non-conductor, which seems designed to prevent an undue lateral waste of the passing current, precisely as the varnished cotton or silk wrapping secures greater certainty of transmission along copper wires. And in striking resemblance to such galvanic wires, every nervous filament is only covered in its passage, and left open at its cerebral and villous extremities. More remarkable still, each filament in every fasciculus or bundle of nerves issuing from the brain, has its own separate theca or membranous coat, as if to prevent confusion in the transmission of the neuro-voltaic currents, and secure, unexpended, their appropriate electro-force to the respective points of distribution. In further accordance with these views, it is an imposing fact that adipose matter, a bad conductor,* in the language of Richerand, "covers and surrounds the extremities of the nerves, diminishing their susceptibility, which is always in an inverse proportion to corpulency"—a beautiful arrangement, and admirably adapted to guard against too rapid an escape of this energizing fluid from the fine filiform extremities of these delicate conductors, and to reserve it for the functional demands of the various organs.

---

*And hence tallow is employed to retain amalgam upon the rubber of our electrical machines.

"In fact," adds this eminent physiologist, "it is observed that persons subject to nervous affections constantly join an extreme leanness to an excessive sensibility," accompanied, too, as our experience authorizes us to add, with functional derangements and general constitutional debility, the electric waste being thus made to exceed the electric supplies necessary for the maintainance of hygienic action. Indeed, the law of insulation, or electro-retention, seems to be preserved throughout the animal economy. The organs of secretion, over which the distinguished Marshall Hall predicted that electricity would exercise an extensive influence, and which has since been so ably and satisfactorily verified by Dr. Wilson Phillip and others, are generally found with non-conducting investments. The liver is surrounded with the membranous and lubricated linings of the abdominal cavity in front, and the oleaginous surface of the viscera and the hepato-gastric omentum in the rear. The kidneys are literally immersed in adipose matter, while the mammary glands are liberally supplied with a thick tunic of the same insulator. These may be regarded as but interesting specimens of that electric precaution which nature has manifested in regard to glandular stuctures generally. The same defensive measures have been adopted for the circulatory system.

As, in the calibre of the sanguiferous tubes, chemical changes and consequent electric evolutions are perpetually going on to supply animal heat, and for other vital purposes, it would seem extremely appropriate that lateral radiation should be prevented by sufficient non-conductors. Now, in accordance with this anticipation, the great central organ, the heart, is always containing blood in a highly positive condition—ever fresh from the re-oxidizing region of the lungs—is encompassed by fatty and membranous textures, while, in the language of the author already quoted, "adeps is found in great abundance in the direction of the blood vessels." Yet with all this delicate conformity to electric law, there must occur in its passage a diffusive expenditure of the fluid, which, it would seem, should be compensated by timely accessions from some adequate source.

In the construction of our telegraphic lines, it is well known to electricians that the length of the wire determines the strength of the battery. As the first is extended, the second must be increased to allow for lateral waste or defective conduction, and if the route be continued beyond a convenient distance, re-lay batteries must be interposed at intervening points to reinforce the waning current.

Now, may not the mysterious ganglionic system, that puzzle of all former physiology, be intended as a series of relay batteries, stationed along the nervous routes to re-supply the partial exhaustion and maintain the normal momentum of the electric forces? And to give plausibility to this suggestion, it must be remembered that these multiplied nervous centres generally occur in the neighborheod of the large secreting organs, and where electro-vital accumulation seems to be most demanded.

It is interestingly true, and to some extent an entirely analagous arrangement, that in the electric organs of the torpedo, which consist of from four to five hundred parallel prisms, each separated by transverse diaphrams with intervening cells, each containing water, albumen and chloride of sodium, the nervous distribution is abundant and peculiar, "forming a great number of closed circles, each of which has one (nerve) in the cerebral lobe, and another in the wall of the cell of the organ."

Indeed, dissections of the torpedo and gymnitus show that the ganglia and nerves of their electrical apparatus exceed in extent all the rest of the nervous system put together. "The analogies," says Matteucci, "between muscular contractions and the discharge of the torpedo are complete; everything which destroys, augments or modifies the one, equally effects the other." The shock which it communicates is decidedly a voluntary act, and so completely under the control of the will, that it can at pleasure discharge either the whole or a part of its organ.

In this class of animals we have, beyond question, the nervous power converted into electric force; and why may not the converse be true, viz., that electric force may be transformed into nervous power? The reciprocal generation of distinctive phenomena, both in the inorganic and organic kingdoms, all clearly traceable to a community of agency, and indicating throughout the presence and control of electric currents, modified only by the organization, the condition or the circumstances of the bodies through which their manifestations are made, may be regarded as settled results in science. To illustrate: In the inorganic universe, electricity produces chemical action, and chemical action, in return, evolves electricity. Galvanism (only another form of the fluid) generates magnetism, and, conversely, magnetism originates powerful galvanic currents. Again, galvanism produces caloric, and caloric reciprocally sets in motion strong thermo-electric currents, capable, even in the laboratory, of producing results identical with those of galvanism, as shocks, sparks and magnetism. But this correlation of physical forces, or convertibility of power, is to be found in the vegetable kingdom also. Plants are now known, through the activity of their vital forces, to throw off immense quantities of electricity during the progress of healthy growth, while electrical action, in return, has exhibited effects almost magical in rapidly developing all the phenomena of vegetable life. Now let us ascend to the more complicated structure of the animal kingdom, and when it is incontestibly established that in the case of electric fishes nervous power is converted into electric force, why should it be regarded either unphilosophical or improbable—nay, are we not constrained to expect that electro-dynamic manifestations should be the result of nervous power? or, in other words, that under the laws of animal life they are convertible forces?

While the Tuscan professor does not consider the *vis nervosa* to be identical with the electric current, he is commendably guarded in detailing the result of his experiments. When attempting to deflect a galvanometer by exciting irritation in the nerve of a living animal, he says, "I must confess that whenever the experiment was well made, I never obtained evident and constant traces of the electric current." Again, in endeavoring to effect the same thing by the aid of a helex, he adds, "I never observed constant signs of the induced current in the spiral." Upon the subject of neuro-electric currents, therefore, we must still regard the affirmative results of the experiments of Dr. Prevost, of Geneva, in 1848, sustained subsequently by those of Prof. Zantideschi and Dr. Favio, and hereafter referred to in this article, as uninvalidated. But it has been objected that when a nerve is ligated, the parts beyond the ligature upon which it is distributed cease to feel or act under the determination of the will, while an electric current, sent along the same nerve, passes to its extremity and exhibits its characteristic effects upon the contractility of the muscular fibre, and that therefore electricity is not identical with the neuro-vital power. Now, it does seem to us that a fair and ingenuous admission of existing laws, and all the facts involved, would very much weaken if not entirely remove the force of this objection, however honestly suggested or honorably maintained. First, then, let it never be forgotten that those physical laws which ordinarily regulate and govern the elements of inorganic matter, are at once modified, suspended or abrogated when those elements are transferred to the dominion of life and made subordinate to its higher control, and the very same agent which, beyond the powers of life, was characterized by certain and unchanging indications, when marshalled within the circle of vitality, is seen under new aspects and often capable of higher results, while its identity remains undoubted. Mark the peculiar and complex combination of the simple substances, oxygen, hydrogen, carbon and nitrogen, in inorganic compounds—the multiplicity of the atoms employed, and the diversity of their chemical aggroupments in constituting such forms of matter as sugar, starch, muscle, tenton, cartilage, etc., etc., and that just as soon as the vital principle has fled and the unrestrained play of inorganic affinities commences, these compounds are suddenly resolved into their elements, which again instantly reunite under new atomic arrangements in the formation of water, carbonic acid, ammonia, etc. Witness, also, the suspension of the ordinary laws of caloric in the uniformity of animal temperature, whether at the equator or the arctic circle—under a heat of 352 degrees Farenheit with the "oven girls of Germany," or in the bleak atmosphere of the mariner in the polar seas at 50 degrees below zero Farenheit.

Now we have only to suppose that vital currents are graduated in their momentum to suit precisely the exceedingly delicate tissue which they are designed to traverse, viz., the pulpy or fluid matter invested by the neuri-lemma, and that the normal electric impulse is too feeble to pass the obstruction and put in motion the molecules beyond, while an artificial current will pass over a breach of continuity, even in wire, to make its circuit.* This fact seems to have modified the views of Prof. Matteucci himself, in his examination of induced currents, and after having ascertained that "very feeble electric discharges," whose presence could not be detected "either by the galvanometer or any other instrument," produced contractions in frogs. He tried such weak discharges from a Leyden jar upon a galvanoscopic frog, and admits that after "a great number of endeavors," he could discover no difference between the inductive effects of such currents and those produced by the normal muscular currents of the animal. Before closing our remarks upon this department of our subject, we cannot forbear calling attention to the very recent anatomical discovery, that the muscular fibres are directly continuous with the tendonous fibres in which they terminate, and that the sarcolemma, or superficial investment of the muscle, stops abruptly short at the junction of the muscle with the tendon. In this arrangement, therefore, we have "some reason to consider the tendon as having the same electrical state as the interior of the muscle, so that by establishing, by means of a good conductor, a circuit between the tendon and the sarcolemma which covers the muscle, we put in circulation a portion of the muscular current already proved to exist."

5th. *The phenomena of electricity are only manifest when the electric eqilibrium is disturbed and then restored by instituting a current between the positive and negative surfaces.*

Now the most recent and thorough investigations in neutrology authorize us to believe in the circulation of electro-vital currents along this sensitive line of conductors, giving strong indications of conformity to the above law of the inorganic fluid. To adopt the language of a distinguished member of the Royal Academy of Medicine in Paris, (Dr. Husson) "the structure and functions of the nervous system have of late become the study of physiologists; and the opinions of Reil, D. Autenreith and M. D. Humbolt, as well as the recent works of M. Bogros, seem to place beyond a doubt not only the

*In connection with the explanation, however, it must be recollected that by Prof. M's experiments on an insulated nerve ligated the current was "weakened and more feeble" in its manifestations below the cord.

existence of a nervous circulation, but also the exterior expansion of this circulating fluid—an expansion which takes place with such force and energy as to create a sphere of action that may be likened to that in which we observe the action of electricity." These views receive further support from the researches of Prof. Zantideschi and Dr. Favio, who assert the existence of two sets of neuro-electric currents—one superficial, and going towards the cerebro-spinal axis, the other more deeply seated, and going from that axis to the surface. Now, should the superficial or returning current not be traceable by any tests now at our control, we are fairly authorized to infer its existence from a recently discovered fact in telegraphic electricity, viz., that it is only necessary to make provision for the outgoing current along a single wire, and by allowing the extremities each to come in contact with the ground, though fifty miles apart the returning current effects its invisible passage along the earth to its point of exit—faithfully completing its electric circuit after having performed the task assigned it. But infallibility to establish the existence, not only of currents, but voltaic currents along the nervous cords, we must refer to a fact which finds its anologue in another law of the fluid in its inorganic manifestations.

6th. *A current passing under given circumstances at an angle of about* 90 *degrees from a soft iron bar, instantly converts it into a magnet.*

The capability of our fluid to generate magnetism is too well known to require illustration here. Now the interesting experiments of Dr. Prevost, of Geneva, as reported by M. De la Rive to the French Academy in '38, clearly settles the existence of such currents, for Dr. P. "succeeded in magnetizing very delicate soft iron needles by placing them near to the nerves and perpendicular to the direction which he supposed the electric current took. The magnetizing took place at the moment when, on irritating the spinal marrow, a muscular contraction was effected in the animal."

7th. *Electricity enters and escapes from pointed conductors with great facility.*

We see an obedience to this law interestingly exemplified in the animal phenomena of taste, absorption, etc. To the pen of that great physiologist, Sir Charles Bell, we are indebted for an accurate and graphic description of these delicate processes, given, it is true, without any apprehension of the conclusions to which his anatomical testimony might lead. He says, "the papillæ, which are the organs of taste, are to be seen on the point and edge of the tongue, and consist of a pretty large, vascular soft point, which projects from an opaque, and white sheath. If we touch one of these papillæ with the point of a brush" (dipped in vinegar) "and at the same time apply a magnifying glass, it is seen to stand erect and rise conspicuously from its sheath, and the acid taste is felt to pass backwards to the root of the tongue." The simple rationale of this process may be thus enunciated, viz., the acid, being a negative electric, excites the positive electricity of the nervous villi, and attraction and erection are the natural consequences, as the applied finger produces the extention of cotton fibres upon a charged prime conductor. Our time forbids the further prosecution of these interesting examinations, and we must only remark that Sir Charles Bell's detail of the apparatus and process of absorption furnishes to our minds satisfactory proof of the electrical character and action of the lacteals and lymphatics. The "lanuginous surface" of the intestines, the "small filaments" which are seen by "a magnifying glass," projecting from the surface "like hairs," when stimulated by the negative contents of the bowels, and then "erected" in the act of absorption—all speak plainly the electric nature of this process.

8th. *Electricity expands fluids without chemical change and increases the velocity of their motion through capillary tubes.*

A fluid passing by drops from a capillary orifice, as soon as electrified by contact with a charged conductor, flows in a fine but rapid and continuous stream, until the communication is broken. May not this agency be the primum mobile of the circulation in the proper capillary tubes, forming the anastomosing net-work between the veins and arteries? for it seems to us, from the minuteness of their calibre and the ordinary density of the blood, it is impossible satisfactorily to account upon any other received hypothesis, for the constant and speedy movement of that fluid in the remote quarters.

9th. *Curved or spherical surfaces retain accumulated electricity best.*

This great law of our fluid is regarded in the cylindrical form and rounded extremities of every prime conductor and the globular knob of every Leyden jar, and yet it is strange that no writer with whom we have met seems to have recognized a conformity to it in the general contour of the human body. The undulating outline and beautiful rotundity of the human figure, have sprung the imaginations of enraptured poets and furnished a theme for adoring lovers; rhetoricians have lectured upon the purity of taste and philosophized upon the "curved line" and the "line of beauty," but it seems never to have caught the observation of poets, lovers or rhetoricians that man was shaped for the material world in which he moved, and in conformity with the great physical laws which control the elements within and around him. Our tastes are given us by a benign Creator to meet the circumstances of our being and administer to the pleasures of life. But had Heaven so constituted these tastes, the pleasing emotions which are now excited by the gentle curve of Beauty's cheek and the graceful ronudness of her sylph-like form, would have been characterized by equal intensity had that cheek stood out a triangle and that form an obelisk.

Was not, then, the great design of our external conformation to give additional security against an excessive discharge of this vitalizing fluid? Surrounded, as we are, on every hand and at all times by an aereal medium, often transformed by suspended moisture into a rapid absorbent of electricity—a fluid so indispensable in its control over the animal functions, special guards seem to have been thrown around this exalted sphere of its action. Besides, the nervous sheaths, the sanguiferous tubes and the oleaginous accompaniments of both, the exposed outposts of the system are fenced in by a general subcutaneous expansion of adipose tissues—itself surmounted by a final and admirably insulating investment, in the squamous cuticle which strictly conforms to electric laws in its curvilinear adaptation to the whole corporeal surface, and is supplied by an oily secretion which, while indispensable to healthy existence, is nevertheless constituted a negative electric to facilitate the accomplishment of the same grand end. Many warm-blooded animals whose position in the scale of being requires that they should be almost constantly immersed in water, as the whale, seal, walrus, etc., are protected against undue electrical exhaustion, and consequently from an abnormal reduction of temperature, even in the conducting medium through which they move, by immense masses of non-conducting unctious matter which surrounded the whole body to a considerable depth under the cutis vera. It is an interesting and relevant fact which chemistry has recently disclosed, that the horny texture of the epidermis is not only in chemical composition nearly the same, but in non-conducting power precisely similar to hoofs, hair, horns, nails, claws, wool, etc., and is completely represented (where, according to our view, its insulating presence is demanded) in the corneous covering of the epithelium which lines the inner surfaces of the blood and lymph vessels, of the intestinal canal and the passages from most glands as well as the mucous membrane of the lungs. The extremities of animal bodies therefore, and those parts approximating most to points, are, in accordance with the same design, guarded by insulators. Hence the beautiful adaptation of the hoofs and horns of quadrupeds, the talons and beaks of birds, etc., to effect the high purposes of Nature.

The very instincts of mankind seem to have led them, in many instances, to the adoption of habits conformable to the action of this law. Nor is this remark limited to civilized more than to savage life. Artificial heat, by drying the air and the cutaneous surface, condnces largely to electrical retention, as well in the rude hut of the Laplander, as the costly parlor of the American. So do the greasy skins, furs and feathers of the Indian tribes, and even the rancid and offensive fish oil with which the New-Hollander besmears his body, while the enlightened civilization of cis-and trans-Atlantic states, affects the same object in the non-conducting textures of the cottons, linens, woolens and silks, with which they are clothed. The same instinctive conformity to the inflexible habitudes of our fluid, may be found in the sleeping arrangements of both savage and civilized life. For while the organic electricity of the Western Indian is preserved upon his buffalo and bear-skins, and that of the wild Polynesian upon his dry grass and mats, the Asiatic reposes electrically safe upon his muslins and silks and hair matresses, and the European and

American upon their cotton stuffs, blankets and feather-beds. In accordance with these views, the health and longevity of our race must be materially affected by the moisture or dryness, and consequent electrical condition of the atmosphere. Hence on the lofty table lands of Mexico, 8,000 feet above the level of the sea, and amid whose pure, and dry, and transparent air, the moon is seen as a sheet of silver, and the stars as burnished gold, embossing a vault of Lapis Lazoli—human life, according to the testimony of Humboldt and Murray, is frequently extended to the long period of "one hundred years, and is still green and vigorous;" while, according to the same testimony, down the slopes extending to the sea, "where humid winds and frequent fogs prevail," and where corporeal electricity is therefore rapidly absorbed and lost, the air is almost fatal to human life. Here we are furnished, too, with a satisfactory explanation of the deleterious consequences resulting from exposure to damp sheets or garments, and especially in the case of invalids or enfeebled constitutions, where the electro-generative forces are weak, and unable to compensate for the waste by absorption—hence functional derangements rapidly supervene.

Suffer us now, after having traced this parallelism of electrical action between inorganic and organic, inanimate and animate matter, briefly to refer to some facts within reach of most observers, illustrative of the control which the electric current exerts over the excitability of the nervous tissue and the functional phenomena dependent upon its action:

1. It increases, under appropriate polar arrangement, the peristaltic motion of the entire intestinal canal.

2. A galvanic line of communication established by a conducting wire and terminal plates, between two slightly excoriated surfaces on the human body, soon presents, under the positive pole, active inflammation, excess of nutrition, and if kept within conservative bounds, a rapid restoration of the parts; while the negative surface indicates an enfeebled vitality, deficiency of nutrition, and an approrch to gangrene and mortification.

3. The circulatory salts of the human body may be decomposed at the cutaneous surface, by a similar arrangement—

the acid collecting at the positive pole and the alkali at the negative—each being indicated by its peculiar characteristics.

4. Neuralgic pains have often been relieved by the passage of a thunder storm—a phenomenon for which a rational solution is only to be found in the fact that the changed electrical condition of the atmosphere had effected a disturbance in the electrical equilibrium of the system, producing, perhaps, abnormal and irregular circulation of the neuro-electric currents, a state of things which was at once relieved, as soon as the atmospheric balance was restored by the discharged clouds.

5. Cases of hemiplegia and other paralytic affections have often found essential relief from the application of electric fluid in some of its forms. Under the generating power of a large electro-magnet and revolving armature I have seen a case of paralysis of the muscles of the eye, cheek, lip, etc., entirely relieved, after a few applications of fifteen minutes each, which had resisted the ordinary treatment for several months previous.

6. Irregular and spasmodic actions in the limbs or joints, dependent, as we suppose, upon the disturbed electro-vital currents which traverse the system, will frequently find a remedy in the use of a similar instrument. My esteemed colleague, in the chair of surgery, has kindly furnished me with a case of spasmodic contraction of the flexor muscles of the knee-joint, which yielded to the first application of a moderate magneto-electric current.

7. The beautiful and careful experiments of Broussais show the control of the electric fluid over the processes of absorption and deposition. He speedily generated a milky cataract in the eye of a pig, by introducing into the crystaline lens a fine copper wire, connected with the negative pole of an active galvanic series, and two weeks afterwards as speedily removed it by reversing the poles.

8. Satisfactory experiments by Dr. Wilson Phillip, and since, I believe, "publicly tested in most of the medical schools of Europe," have shown that respiration and digestion can, for some time, be carried on after the bi-section of the pneumogastric nerve, which so largely controls these functions, by sending a positive electric current through the pulmonary and gastric segments.

From our acquaintance, then, with an agent so prompt and powerful in its control over the sensibilities and functions of the animal organism, may we not, hereafter be able intelligibly to trace the *methodus medendi* of many remedies, and the rationale of certain pathological phenomena heretofore enveloped in perfect mystery? Does not *acu-puncturation*, formerly employed more extensively than at present, in cases of cephalagia and rheumatism, owe its curative efficacy to the conducting power of the inserted needles, which convey silently off the excess of electricity collected in the part? Again, may not the etiology of malarious and contagious diseases, which has long lain in profound and unapproachable obscurity, be more satisfactorily explained by admitting the agency of electrical affinities than upon any other hypothesis? The human blood, in a normal and healthy condition of the system, is in a positive state, which is constantly maintained by the activity of the generating sources within—an excess generally passing off in silence from the cuticular surface, so that out of 356 experiments made by Mr. Hemmer, of England, upon the "uncovered skin," 322 indicated the presence of positive electricity. This surplusage of the fluids upon the surface, we believe to be indispensable to the healthful condition of the whole animal economy, and that when, from any cause, it is diminished or ceases, diseased action ensues. I have just received an interesting communication from Mr. D. Ruggles, a blind man, of Northampton, Massachusetts, who has, for years, been able to determine upon the character of diseases from the electrical manifestation furnished by the skin to his acute sense of touch. "These symtoms," says Mr. R. in his letter to me, "vary in healthy persons and in invalids; in the former I can feel a regular emission of what I believe to be electricity, from the pores of the skin in any part of the body, and especially near the wrists; in the latter this symptom is feeble and often intermitent, and in some individuals it is not perceptible." He then gives in detail the peculiar electrical states exhibited in several different diseases, as detected by sensation.

Now, by the testimony of the best authorities to which we can have access, it may be regarded as certain that the pestilential exhalations which escape from animal putrefactions, vegetable decompositions, etc., and, indeed, all the ascertained causes of epidemic and infectious diseases, are in an electro-negative condition, and when the human body is surrounded by a highly charged negative atmosphere, instead of the naturally positive state of dry and pure air, the result must be the rapid absorption and exhaustion of the positive corporeal supplies, until the electro-vital resistance is overcome, and functional aberrations supervene. May we not in this way largely account for the ennui and lassitude so commonly experienced in a damp atmosphere, where the conducting power of the medium too rapidly carries off the organic currents, and thus superinduces a general enervation, the barometrical column, in the mean time, indicating too little reduction in atmospheric pressure to have produced such deleterious effects upon the animal sensibilities?

Once more: we believe that the true pathological distinction between the chronic and acute forms of rheumatism will be found to depend upon the electro-negative condition of the parts in the former, and their electro-positive states in the latter—the curative applications plainly appropriate in the one, being contra-indicated in the other. The high inflammatory action, arterial turgescence, florid surface, and great pain in acute articular rheumatism, giving evidence of the positive fluid, which is but increased by stimulating applications, warm covering etc., and attended by an aggravation of the prevailing symptoms, and which can only be successfully treated by withdrawing a portion of blood, (electro-positive) by venesection or otherwise, the application of negative-electrics, as vinegar, anodyne lotions, etc., and thin investments around the joint; or, in short, by such remedies as will discharge the excess of the positive fluid. Whereas in the chronic form, the absence of capillary engorgement, heat, redness, etc., and an abatement of pain from warm covering, indicate a minus condition of the part, and require that the electric equilibrium should be restored by favoring the accumulation of the positive fluid, by warm poultices, flannel or silk bandages, or other non-conductors. In accordance with these views, therefore, in acute rheumatism, or an electro-positive condition of the part, allow us respectfully to suggest the use of a flexible metalic bandage, or strips of tin-foil upon linen, studded thickly with metalic points, directed outward, which may be readily prepared by driving small tacks through the bandage, and applying their heads towards the cutaneous surface, keeping the whole moist, in the mean while, by embrocations of tobacco and vinegar, or other negative electric.

Lastly: The singular phenomena attendant upon etherization, or the use of trichloride of formyle, (chloroform) which belongs to the same class of volatile fluids, for which we have yet seen no plausible philosophical solution, may be fairly traced, we think, to the electrical affinities at play in the pulmonary organs and the sanguiferous channels, and directly affecting the whole nervous tissue. The views here inculcated, it must be remembered, recognize all sensation and motion, as dependent upon nervous energy, and the latter as only maintained by the presence of electric currents. Now we have tested by repeated experiments, that the vapor of sulphuric ether is always negative in the presence of a positively excited body, and when freely inhaled it posses readily by inhibition, through the mucous lining of the air-cells into the circulating mass of blood, whose normally positive condition is rapidly reduced, until the supply is inadequate to maintain the integrity of functional action in the sensiferous nerves, and consequent insensibility follows. If the inhalation is continued, the motor system of nerves next lose the necessary amount of electrical stimulus, and the whole muscular tissue is relaxed, and voluntary motion suspended; and, if persevered in too long, the involuntary motion of the heart, arteries, etc., cease from the same cause, and death ensues. When, however, moderately administered, spontaneous restoration may be expected from a re-supply of the positive fluid, furnished by the still active electro-motive resources of the system.

Some experiments recently made by M. Ducros for the purpose of determining the efficacy of electricity in recovering inferior animals from the effects of inhaled ether, and published in the August number of the London *Lancet* greatly strengthen our opinions as to the rationale of letheonization. M. Ducros states, " that the phenomena produced by the vapor of ether on chickens and pigeons last from seven to eight minutes; but if these birds, when thus under the influence of ether, are submitted to the action of electricity by placing them on the isolating stool, and allowing a current of positive electricity to pass into them, they recover from their insensibility in about thirty seconds. If they are placed on the electric conductor, they are aroused in ten seconds; and if they are placed at the extremities of the conductors, and thus made to receive electric shocks, their vigor is instantly restored. When submitted to the action of the simple apparatus of Clarke, the electro-magnetic current is equally instantaneous in effecting their restoration. The influence of the negative electricity, instead of abridging the insensibility, appears to prolong it."—*Comptes Rendus and Medical Gazette.*

